# AKR1B10 accelerates glycolysis through binding HK2 to promote the malignant progression of oral squamous cell carcinoma

**DOI:** 10.1007/s12672-024-00996-0

**Published:** 2024-04-26

**Authors:** Ye Cai, Huiling Li, Diya Xie, Yanan Zhu

**Affiliations:** 1 Department of Endodontics, Nanjing Stomatological Hospital, Affiliated Hospital of Medical School, Research Institute of Stomatology, Nanjing University, 30 Zhongyang Road, Nanjing, Jiangsu 210008 People’s Republic of China; 2 Department of Oral Pathology, Nanjing Stomatological Hospital, Affiliated Hospital of Medical School, Research Institute of Stomatology, Nanjing University, Nanjing, Jiangsu 210008 People’s Republic of China; 3 Department of Oral and Maxillofacial Surgery, Nanjing Stomatological Hospital, Affiliated Hospital of Medical School, Research Institute of Stomatology, Nanjing University, Nanjing, Jiangsu 210008 People’s Republic of China

**Keywords:** AKR1B10, Epithelial mesenchymal transition, Glycolysis, HK2, Metastasis, Oral squamous cell carcinoma

## Abstract

**Background:**

Oral squamous cell carcinoma (OSCC) remains a rampant oral cavity neoplasm with high degree of aggressiveness. Aldo–keto reductase 1B10 (AKR1B10) that is an oxidoreductase dependent on nicotinamide adenine dinucleotide phosphate (NADPH) has been introduced to possess prognostic potential in OSCC. The present work was focused on specifying the involvement of AKR1B10 in the process of OSCC and its latent functional mechanism.

**Methods:**

AKR1B10 expression in OSCC tissues and cells were detected by RT-qPCR and Western blot analysis. CCK-8 method, EdU staining, wound healing and transwell assays respectively assayed cell viability, proliferation, migration and invasion. Immunofluorescence staining and Western blot evaluated epithelial mesenchymal transition (EMT). Adenosine triphosphate (ATP) contents, glucose consumption and extracellular acidification rate (ECAR) were measured by relevant commercially available kits and Seahorse XF96 Glycolysis Analyzer, severally. The expressions of proteins associated with metastasis and glycolysis were examined with Western blot. Co-IP assay confirmed the binding between AKR1B10 and hexokinase 2 (HK2).

**Results:**

It was observed that AKR1B10 expression was increased in OSCC tissues and cells. After AKR1B10 was knocked down, the proliferation, migration, invasion and EMT of OSCC cells were all hampered. Additionally, AKR1B10 silencing suppressed glycolysis and bound to HK2 in OSCC cells. Up-regulation of HK2 partially abolished the hampered glycolysis, proliferation, migration, invasion and EMT of AKR1B10-silenced OSCC cells.

**Conclusion:**

To sum up, AKR1B10 could bind to HK2 to accelerate glycolysis, thereby facilitating the proliferation, migration, invasion and EMT of OSCC cells.

## Introduction

Squamous cell carcinoma of the head and neck that occurs in the oral cavity, oropharynx, larynx or hypopharynx constitutes the sixth most prevalent cancer on the global scale [1]. Oral squamous cell carcinoma (OSCC) that may be initiated by tobacco and alcohol use, betel quid chewing and viral infections forms the majority of all oral neoplasm cases [1–3]. According to the Global cancer statistics in 2020, there are over 370,000 new OSCC cases and 170,000 death cases annually [4]. The primary symptoms are oral dysfunction including chewing and swallowing disorders, profoundly impacting patients’ life quality [5]. In spite of the existing therapeutic options represented by surgery combined with radiotherapy and chemotherapy and emerging newly developed treatment options such as immunotherapy, gene therapy and bionic technology [6, 7], the prognosis of OSCC patients remains dismal attributed to high rates of local invasion and metastasis [8]. With the aim of identifying biomarkers to effectively hinder the process of OSCC, it is imperative to figure out the molecular basis underpinning the development of OSCC.

Aldo-ketoreductase family 1 member B10 (AKR1B10, also known as ARL-1), a member of the human aldo–keto reductase (AKR) superfamily, is known as a cytosolic nicotinamide adenine dinucleotide phosphate (NADPH)-dependent oxidoreductase enzyme that metabolizes a variety of endogenous compounds including carbohydrates, steroids, prostaglandins and exogenous carbonyl compounds [9]. A growing body of literature has revealed the dysregulated AKR1B10 expression in a wide range of human malignancies, such as breast cancer [10], gastric cancer [11], colon cancer [12], and so on. Importantly, increasing evidence has uncovered that AKR1B10 expression is elevated in OSCC tissues and associated with tumor recurrence and poor prognosis [13–15]. However, the latent molecular mechanism of AKR1B10 in the development of OSCC is still unclear.

The abnormality of energy metabolism is a common event during the process of cancers, in which, aerobic glycolysis (as known as Warburg effect) provides the main source of energy for tumor cells [16]. Hexokinase 2 (HK2) is one of the hexokinase (HK) isozymes that catalyze the first committed step in glucose metabolism by phosphorylating glucose [17]. Accumulative studies have clarified that HK2 is extensively involved in cancer metabolism and therapeutics [18, 19]. HK2 has been recognized to drive cell growth, metastasis and glycolysis in OSCC, in particular [20–22]. Further, previous evidence has uncovered that AKR1B10 may also participate in glycolysis in hepatocellular carcinoma and cholangiocarcinoma [23, 24]. Intriguingly, AKR1B10 was predicted to bind to HK2 based on the Monarchinitiative database (https://monarchinitiative.org/). Accordingly, it was speculated that AKR1B10 might bind HK2 to function in OSCC through mediating glycolysis, which was also the focus of our present study.

## Materials and methods

### Clinical samples

A total of 15 OSCC tissues and adjacent normal tissues were harvested from surgery patients at the Department of Oral and Maxillofacial Surgery, Nanjing Stomatological Hospital, Medical School of Nanjing University. No patients had undergone radiotherapy or chemotherapy prior to surgery. This study was approved by the Ethics Committee of Nanjing Stomatological Hospital, Medical School of Nanjing University, and with the 1964 Helsinki declaration and its later amendments or comparable ethical standards. All patients provided written informed consent.

### Cell culture

Normal human oral epithelial cells (HOEC; Mlbio, Shanghai, China) was maintained in Roswell Park Memorial Institute (RPMI)-1640 medium (Biosera, Nuaille, France). Human OSCC cells (SCC-4 and SCC-15) procured from American Type Culture Collection (ATCC, Rockville, MD, USA) and human OSCC HSC-3 cells supplied by Mlbio (Shanghai, China) were all cultivated in Dulbecco's Modified Eagle Medium (DMEM)/F12 medium (Biosera, Nuaille, France). All mediums were under humidified conditions with 5% CO_2_ at 37 °C, along with 10% FBS (Biosera, Nuaille, France) as supplements.

### Transfection

The small interfering RNAs (siRNAs) for AKR1B10 that were designated as siRNA-AKR1B10-1/2 (AKR1B10-1, 5ʹ-TGCCTATGTCTATCAGAATGAAC-3ʹ; AKR1B10-2, 5ʹ-TCCTCTCATTTGGAAGACTATCC-3ʹ) and HK2 overexpression plasmid (Ov-HK2) were synthesized by GenePharma (Shanghai, China), setting the nonspecific siRNA (siRNA-NC) and the empty vector (Ov-NC) as the negative control, severally. As per the instructions of Lipofectamine 2000 Reagent (Invitrogen, Carlsbad, USA), the aforementioned siRNAs and plasmids were transfected into HSC-3 cells.

### Reverse transcription-quantitative PCR (RT-qPCR)

Total RNA was extracted from OSCC tissues, adjacent normal tissues and OSCC cells by NucleoSpin® RNA isolation kit (Takara, Dalian, China). The PrimeScript RT reagent kit (Takara, Dalian, Japan) was used to synthesize the cDNA according to the instructions provided by the manufacturer. Target genes were amplified using SYBR Green PCR Master Mix Reagents (Takara, Toyobo, Japan) on the ABI 7500 Real-Time PCR system (Applied Biosystems, Foster City, CA). The relative gene expression was determined with 2^−△△CT^ method [25]. Amplification of β-actin was used as an internal control. The primer sequences for PCR are listed: AKR1B10, forward: 5ʹ-TCAGAATGAACATGAAGTGGGG-3ʹ, reverse: 5ʹ-TGGGCCACAACTTGCTGAC-3ʹ; β-actin, forward: 5ʹ-GCCTCGCCTTTGCCGAT-3ʹ, reverse: 5ʹ-AGGTAGTCAGTCAGGTCCCG-3ʹ.

### Western blot

With the application of RIPA lysis buffer (MedChemExpress, USA) and BCA Protein Assay Kit (Beyotime, Shanghai, China), the collection and quantification of total protein were successively performed. The protein samples were adjusted to the same amount (30 µg/lane) and subjected to 10% SDS-PAGE. The proteins were transferred to the membranes which were then immersed in 5% BSA and treated with primary antibodies against AKR1B10 (cat. no. ab192865; 1/1000; Abcam), HK2 (cat. no. ab209847; 1/1000; Abcam), matrix metallopeptidase 2 (MMP2; cat. no. ab181286; 1/1000; Abcam), matrix metallopeptidase 9 (MMP9; cat. no. ab76003; 1/1000; Abcam), Vimentin (cat. no. ab16700; 1/1000; Abcam), Slug (cat. no. ab302780; 1/1000; Abcam), pyruvate kinase M2 (PKM2; cat. no. ab85555; 1/1000; Abcam), lactate dehydrogenase A (LDHA; cat. no. ab52488; 1/5000; Abcam), E-cadherin (E-cad; cat. no. 20874-1-AP; 1/10000; Proteintech), N-cadherin (N-cad; cat. no. A0433; 1/1000; ABclonal) and β-actin (cat. no. ab8227; 1/5000; Abcam) at 4 °C overnight. Then, these membranes were incubated with HRP‐conjugated secondary antibody (cat. no. ab205718; 1/2000; Abcam) for 1 h. The bands were visualized using an ECL detection reagent (Millipore, Burlington, MA, USA). Densitometry analysis was conducted using ImageJ software (version 1.49; National Institutes of Health).

### Cell counting kit-8 (CCK-8)

After indicated transfection, HSC-3 cells (5 × 10^3^ cells/well) were dispensed into the 96-well plates, each well of which was mixed with CCK-8 working solution (Abmole Bioscience, Shanghai, China) at 37 °C for 2 h in conformity with the recommendations. Under a microplate reader (Ladsystems, Helsinki, Finland), the optical density was surveyed at 450 nm for each sample.

### 5-Ethynyl-2’-deoxyuridine (EdU) staining

The EdU staining was employed to evaluate cell proliferation. 50 µM EdU working solution was added into HSC-3 cells in the 96-well plate for incubation for 2 h. Following the 30 min of immobilization with 4% paraformaldehyde and 10 min of permeation with 1% Triton X-100, cells were dyed by 4ʹ,6-diamidino-2-phenylindole (DAPI). Images were captured under a fluorescence microscopy (Olympus, Tokyo, Japan).

### Wound healing assay

After transfected HSC-3 cells inoculated in 6-well plates (4 × 10^5^ cells/well) were grown at 80% convergence, a wound was formed through scraping with a conventional pipette tip in the cell surface. Under a light microscope (Olympus, Tokyo, Japan), cell movement was documented.

### Transwell assay

For invasion assay, Matrigel (Sigma, St. Louis, MO, USA) was uniformly applied on the surface of the upper compartment of Transwell chambers (Corning-Costar, Cambridge, MA, USA), to which 200 μL cell suspension (DMEM/F12 medium deprived of serum) containing transfected HSC-3 cells (1 × 10^5^) was supplemented. The bottom chamber was stocked with 500 μL medium decorated by 10% FBS. 24 h later, cells on the upper surface were swabbed off. The cells adhering to the lower membrane of the chambers were counted under a light microscope after treatment with paraformaldehyde fixation solution (4%) for 20 min and crystal violet staining solution (0.1%) for 15 min.

### Immunofluorescence staining

After indicated transfection, HSC-3 cells were soaked in paraformaldehyde fixation solution (4%) and permeation agent (containing 0.2% Triton X-100), and sealed in 5% BSA. Subsequently, the cells were treated by primary antibodies that recognize E-cad (cat. no. ab308347; 1/500) and N-cad (cat. no. ab18203; 1/500) from Abcam overnight at 4 °C and Alexa-488-conjugated secondary antibody (cat. no. ab150077; 1/200; Abcam) for 1 h at room temperature. The nuclei were labelled with 5 µg/ml DAPI for 5 min. Images were observed under a fluorescence microscope (Olympus, Tokyo, Japan).

### Co-immunoprecipitation (Co-IP)

According to the standard protocols of Co-IP kit (Invent Biotechnologies), Co-IP assay was conducted. Briefly, 2 μg of antibodies against AKR1B10 (cat. no. NBP1-44998; Novus Biologicals) and HK2 (cat. no. ab209847; Abcam) or goat anti-rabbit IgG (cat. no. 8726; Cell Signaling Technology) were added to HSC-3 cell lysates (containing 2 × 10^7^ cells). The immune complexes were subjected to SDS-PAGE and analyzed by Western blot. HRP‐conjugated secondary antibody (cat. no. ab205718; 1/2000; Abcam) was used in Co-IP western blot assay.

### Evaluation of extracellular acidification rates (ECAR)

With the application of Seahorse XF Glycolysis Stress Test Kit (Agilent Technology, MA, USA), ECAR was measured. Briefly, after indicated transfection, 5 × 10^4^ HSC-3 cells were injected into the 96-well cell culture XF microplates (Agilent Technology, MA, USA), after which the cartridge was sequentially loaded with 10 mM glucose, 1 μM oligomycin, and 100 mM 2-deoxy-glucose (2-DG). Eventually, the results were analyzed by Seahorse Bioscience XF96 Extracellular Flux Analyzer.

### Detection of glucose consumption and adenosine triphosphate (ATP) level

With the employment of ATP assay kit (Nanjing Jiancheng Bioengineering Institute, Nanjing, China) and glucose assay kit (Rongsheng Biotechnology), ATP content and glucose consumption normalized to 1 × 10^5^ cells were respectively determined.

### Statistical analyses

All experiments were repeated three times independently and data are expressed as mean ± standard deviation (SD) and analyzed with GraphPad 8.0 software (GraphPad Software Inc., USA). Student’s t-test was used to analyze the difference between two groups. One-way ANOVA with a post-hoc Tukey's test was carried out to compare difference among multiple groups. P-values less than 0.05 were deemed significant in statistics.

## Results

### AKR1B10 is highly expressed in OSCC tissues and cells and AKR1B10 down-regulation suppresses the proliferation of OSCC cells

**Fig. 1:**
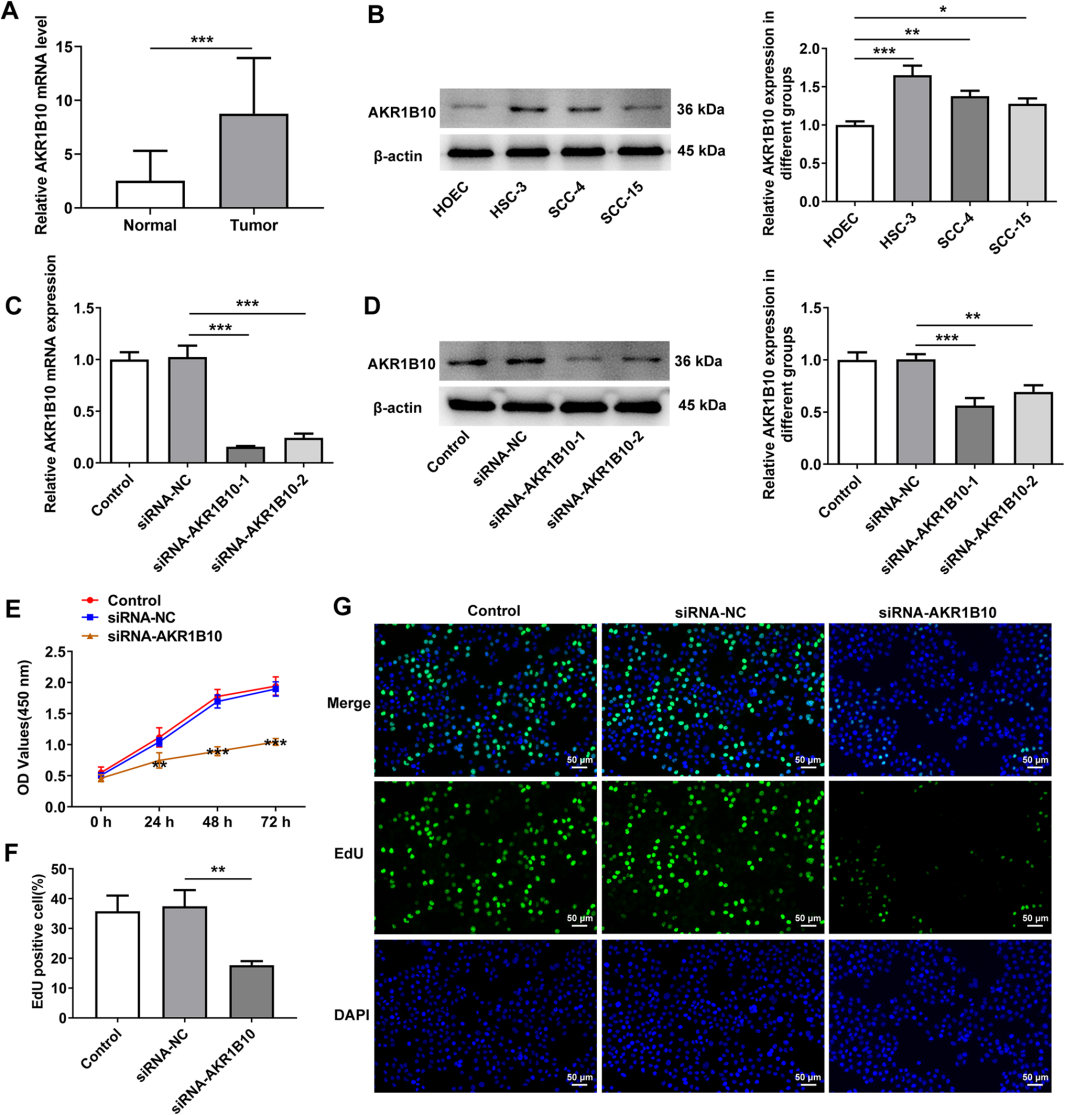
AKR1B10 down-regulation suppresses the proliferation of OSCC cells. **A** AKR1B10 expression in OSCC tissues and adjacent normal tissues was detected by RT-qPCR. **B** Western blot analyzed AKR1B10 expression in different groups (HOEC HSC-3, SCC-4 and SCC-15 cells). Transfection efficacy of siRNA-AKR1B10-1/2 in HSC-3 cells was examined with **C** RT-qPCR and **D** Western blot. *P < 0.05, **P < 0.01, ***P < 0.001. **E** CCK-8 assay appraised the viability of HSC-3 cells. **P < 0.01, ***P < 0.001 vs. siRNA-NC. **F**, **G** EdU staining appraised the proliferation of HSC-3 cells. **P < 0.01

With the purpose of studying the role of AKR1B10 in OSCC, AKR1B10 expression in OSCC tissues and adjacent normal tissues was detected. As shown in Fig. 1A, AKR1B10 was significantly upregulated in OSCC tissues compared with that in the normal group. Additionally, AKR1B10 expression was discovered to be notably increased in OSCC cells (HSC-3, SCC-4 and SCC-15) relative to normal human oral epithelial cell line HOEC (Fig. 1B). Accordingly, HSC-3 cells in which AKR1B10 displayed the highest expression were selected for the follow-up experiments. And the untreated HSC-3 cells were used as the Control group. Prior to the loss-of-function experiments, AKR1B10 expression was distinctly depleted after transfection of siRNA-AKR1B10-1/2 (Fig. 1C, D). The lower AKR1B10 expression was observed in the HSC-3 cells transfected with AKR1B10-1. Therefore, AKR1B10-1 was used to perform the subsequent experiments. As Fig. 1E depicted, the viability of HSC-3 cells was reduced after AKR1B10 was silenced. Further, the results from EdU staining assay illustrated that interference with AKR1B10 resulted in the decline on the number of EdU-positive cells (Fig. 1F, G). Anyway, knockdown of AKR1B10 may play the inhibitory role in OSCC cell proliferation.

### AKR1B10 down-regulation attenuates the migration, invasion and epithelial mesenchymal transition (EMT) of HSC-3 cells

**Fig. 2:**
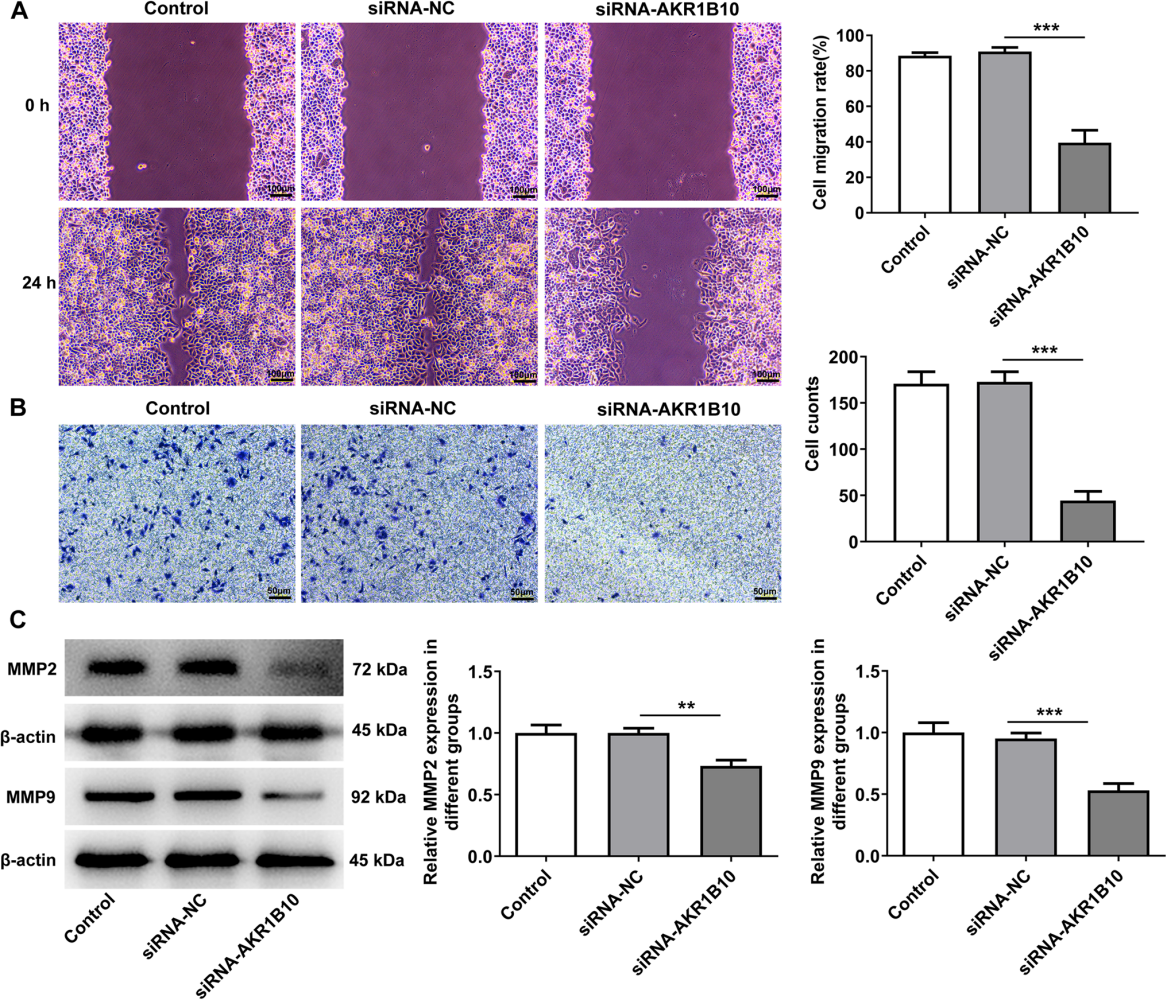
AKR1B10 down-regulation attenuates the migration and invasion of HSC-3 cells. **A** Wound healing assay measured cell migration. **B** Transwell assay estimated cell invasion. **C** Western blot analyzed MMP2 and MMP9 expression. **P < 0.01, ***P < 0.001

**Fig. 3:**
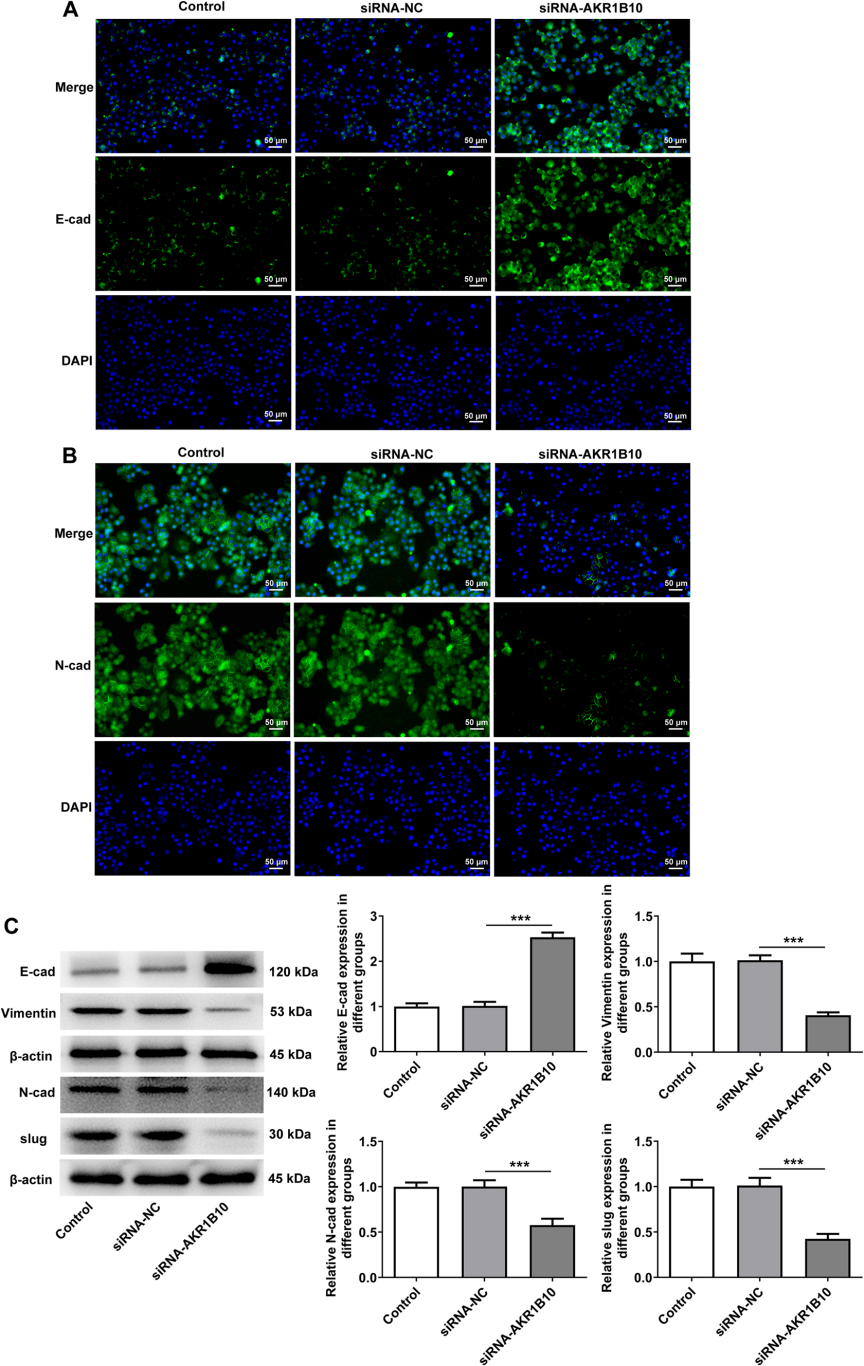
AKR1B10 down-regulation attenuates the epithelial mesenchymal transition (EMT) of HSC-3 cells. Immunofluorescence staining determined **A** E-cadherin and **B** N-cadherin expression. **C** Western blot analyzed E-cadherin, N-cadherin, Vimentin and Slug expression. ***P < 0.001

To further specify the impacts of AKR1B10 on the development of OSCC, HSC-3 cell migration and invasion were also estimated. Through wound healing assay, it was observed that the migration rate of HSC-3 cells was remarkably decreased after AKR1B10 was knocked down (Fig. 2A). Besides, the experimental data from transwell assay expounded that the number of invasive cells was notably on a downward trend when AKR1B10 was down-regulated (Fig. 2B). Also, the protein expression of metastasis-related MMP2 and MMP9 were both lowered by inhibition of AKR1B10 (Fig. 2C). Moreover, EMT is a considered as a requisite for migration and invasion. As illustrated in Fig. 3A, B, Immunofluorescence staining demonstrated that depletion of AKR1B10 elevated E-cadherin expression and decreased N-cadherin expression. Western blot analysis also implied that AKR1B10 knockdown resulted in the up-regulation on E-cadherin expression and the down-regulation on N-cadherin, Vimentin and Slug expression (Fig. 3C). To sum up, inhibition of AKR1B10 may restrain OSCC cell migration, invasion and EMT.

### AKR1B10 knockdown represses glycolysis in HSC-3 cells

**Fig. 4:**
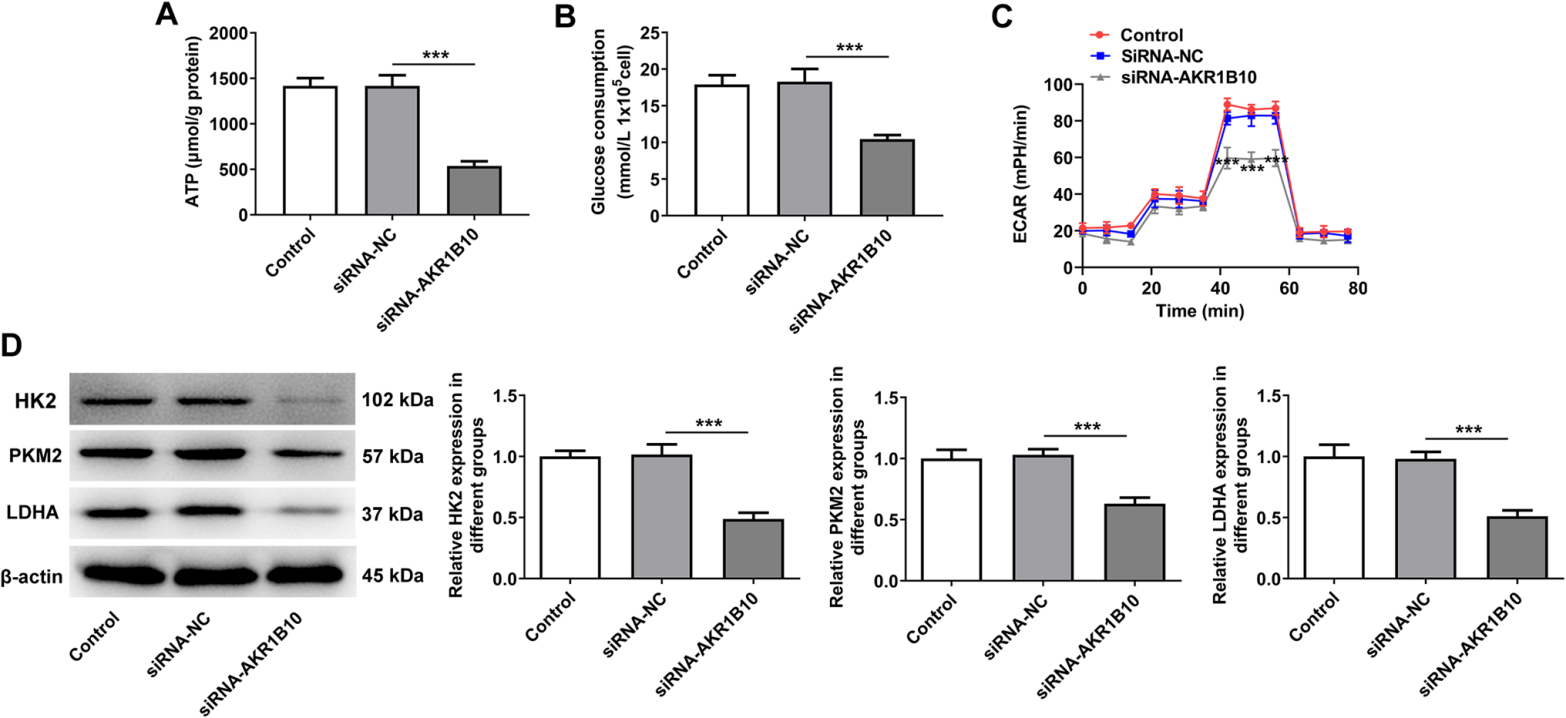
AKR1B10 knockdown represses glycolysis in HSC-3 cells. **A** Relevant kits determined ATP content. **B** Relevant kits determined glucose consumption. ***P < 0.001. **C** Detection of ECAR by Seahorse XF96 Glycolysis Analyzer. ***P < 0.001 vs. siRNA-NC. **D** Western blot analyzed HK2, PKM2 and LDHA expression. ***P < 0.001

Glycolysis has been supported to be related to the progression of OSCC. Therefore, the influences of AKR1B10 on glycolysis in HSC-3 cells were also determined. Notably, insufficiency of AKR1B10 markedly suppressed ATP production and glucose consumption (Fig. 4A, B). Additionally, after AKR1B10 was knocked down, the ECAR was evidently decreased in HSC-3 cells (Fig. 4C). It was also discovered that the expression of glycolysis-related HK2, PKM2 and LDHA were all depleted by absence with AKR1B10 by means of Western blot (Fig. 4D). Collectively, AKR1B10 may contribute to glycolysis in OSCC cells.

### AKR1B10 binds HK2 to accelerate the glycolysis and proliferation of HSC-3 cells

**Fig. 5:**
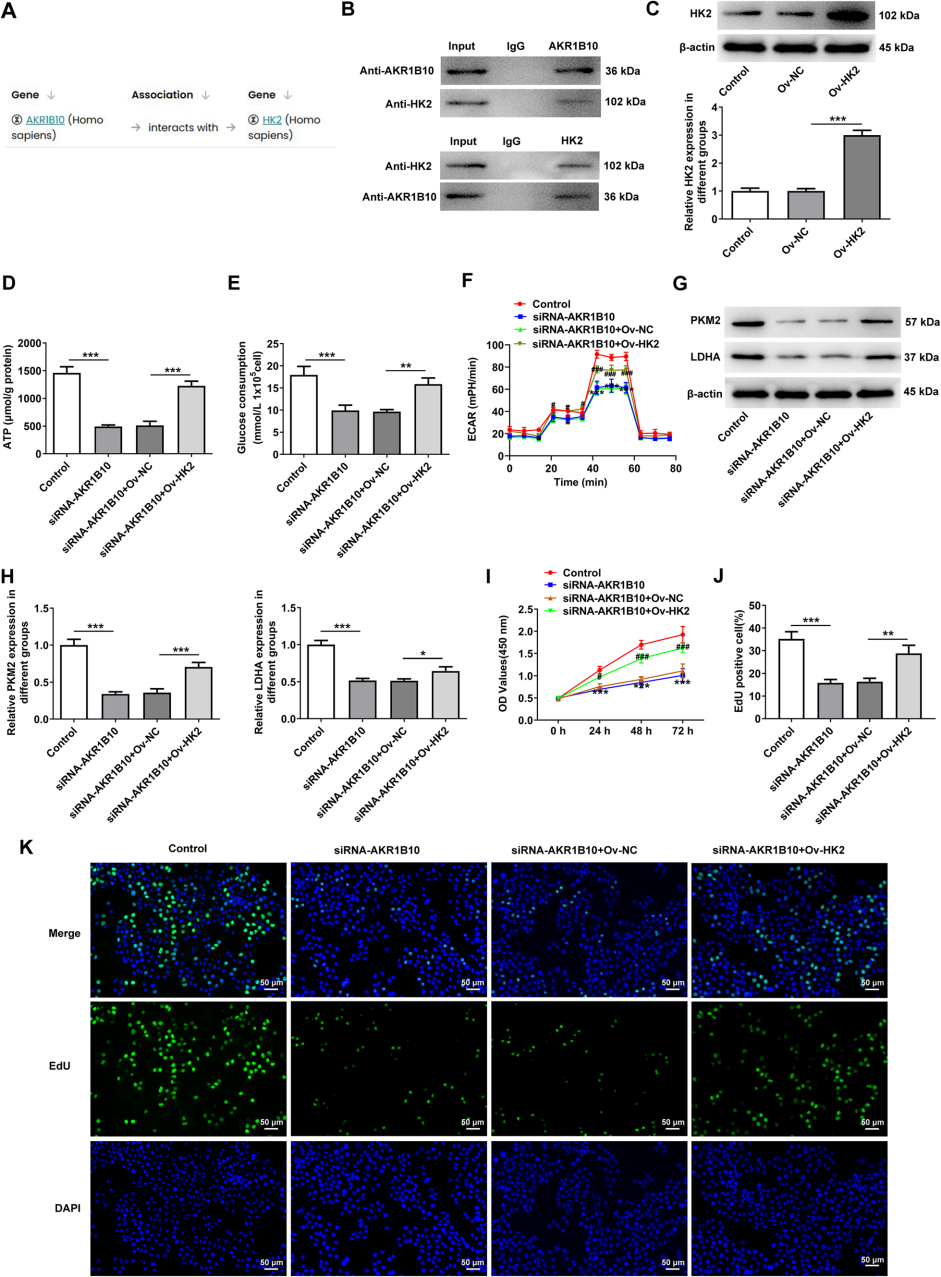
AKR1B10 binds HK2 to accelerate the glycolysis and proliferation of HSC-3 cells. **A** Monarchinitiative database predicted that AKR1B10 could bind to HK2. **B** Co-IP assay testified the binding between AKR1B10 and HK2. Anti-AKR1B10 and anti-HK2 were the antibodies used for western blot, and “IgG”, “AKR1B10” and “HK2” were the IPs. **C** Western blot examined the transfection efficacy of HK2 plasmid. ***P < 0.001 vs. Ov-NC. **D** Relevant kits determined ATP content. **E** Relevant kits determined glucose consumption. **P < 0.01, ***P < 0.001. **F** Detection of ECAR by Seahorse XF96 Glycolysis Analyzer. *P < 0.05, ***P < 0.001 vs. Control; #P < 0.05, ###P < 0.001 vs. siRNA-AKR1B10 + Ov-NC. **G**, **H** Western blot analyzed PKM2 and LDHA expression. *P < 0.05, ***P < 0.001. **I** CCK-8 assay appraised cell viability. ***P < 0.001 vs. Control; #P < 0.05, ###P < 0.001 vs. siRNA-AKR1B10 + Ov-NC. **J**, **K** EdU staining appraised the proliferation of HSC-3 cells. **P < 0.01, ***P < 0.001

Based on the Monarchinitiative database (https://monarchinitiative.org/), AKR1B10 was predicted to bind to HK2 (Fig. 5A). Through Co-IP assay, the binding between AKR1B10 and HK2 in HSC-3 cells was also substantiated (Fig. 5B). To corroborate whether AKR1B10 functioned in OSCC cells via mediating HK2, HK2 expression was enhanced after transfection of Ov-HK2 (Fig. 5C). As expected, the repressed ATP generation and glucose consumption imposed by AKR1B10 reduction were both restored after HK2 was overexpressed (Fig. 5D, E). As portrayed in Fig. 5F, HK2 elevation improved the ECAR which was declined in AKR1B10-silencing HSC-3 cells. Further, AKR1B10 deficiency greatly decreased PKM2 and LDHA expression, which was abated after transfection of Ov-HK2 (Fig. 5G, H). Meanwhile, the diminished proliferation of HSC-3 cells on account of AKR1B10 knockdown was facilitated again when HK2 was up-regulated (Fig. 5I–K). All these findings underline that AKR1B10 may bind to HK2 and HK2 up-regulation partially reverses the suppressive role of AKR1B10 knockdown in the glycolysis and proliferation of OSCC cells.

### AKR1B10 binds HK2 to drive the migration, invasion and EMT of HSC-3 cells

**Fig. 6:**
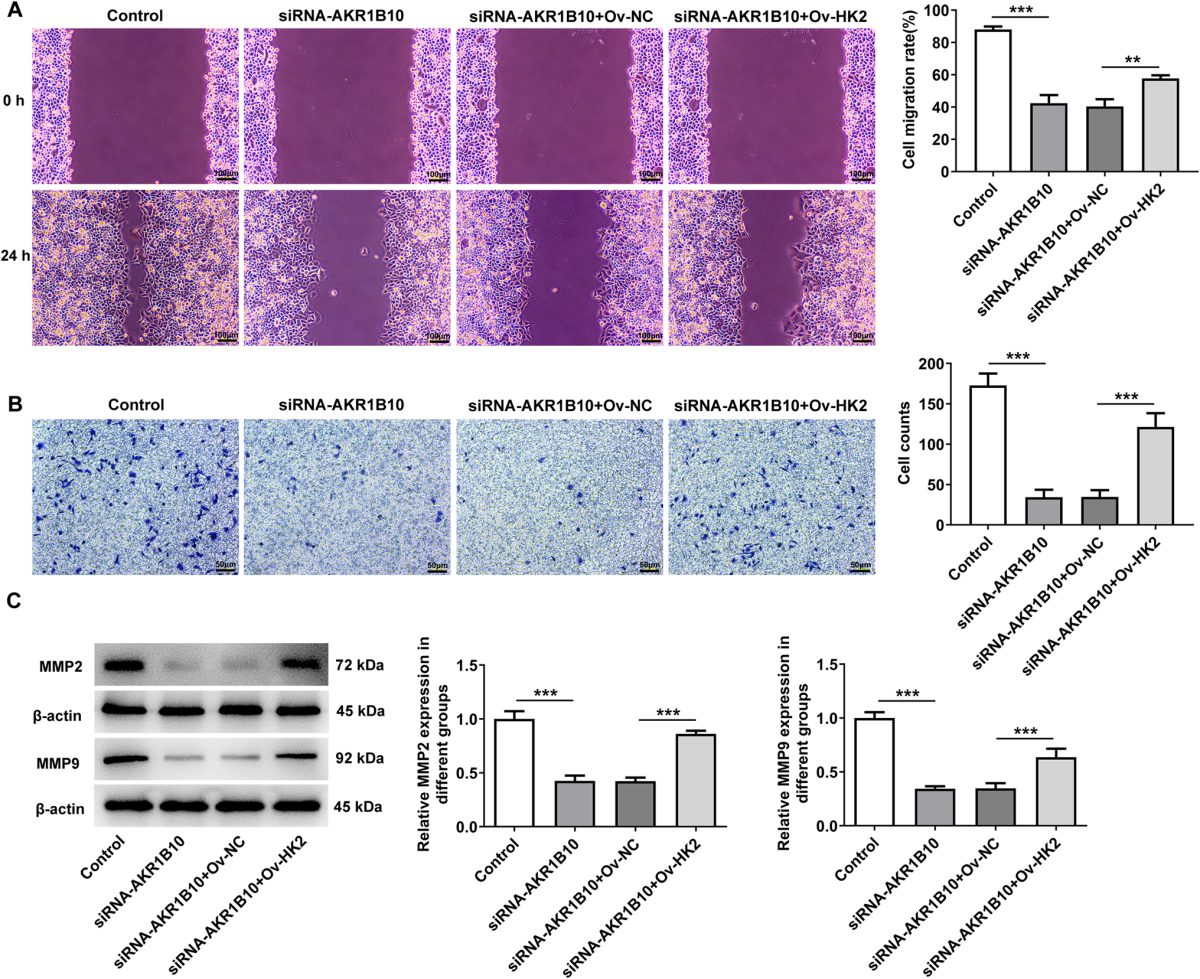
AKR1B10 binds HK2 to drive the migration and invasion of HSC-3 cells. **A** Wound healing assay measured cell migration. **B** Transwell assay estimated cell invasion. **C** Western blot analyzed MMP2 and MMP9 expression. **P < 0.01, ***P < 0.001

**Fig. 7:**
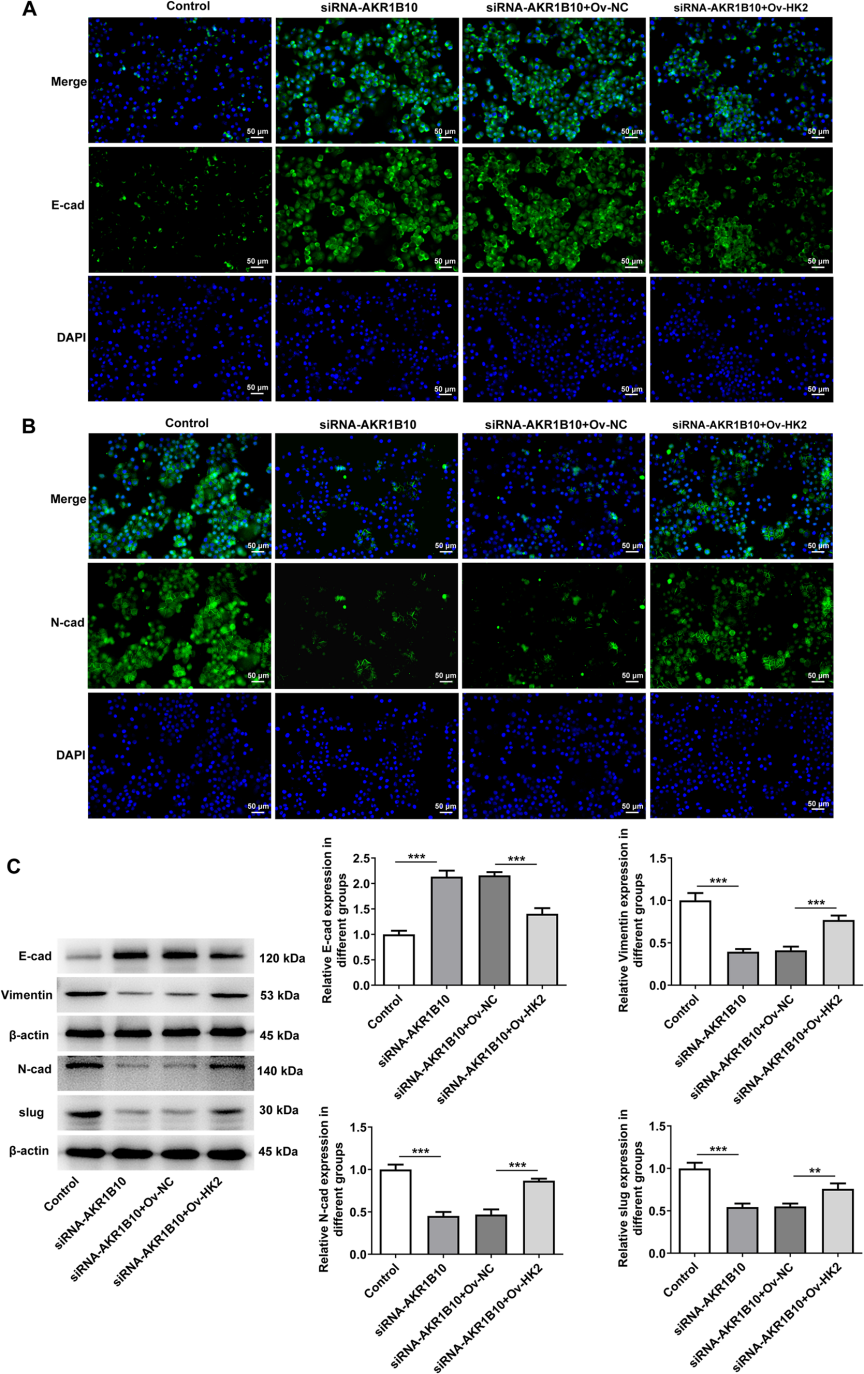
AKR1B10 binds HK2 to drive the EMT of HSC-3 cells. Immunofluorescence staining determined **A** E-cadherin and **B** N-cadherin expression. **C** Western blot analyzed E-cadherin, N-cadherin, Vimentin and Slug expression. **P < 0.01, ***P < 0.001

Concurrently, the results of wound healing and transwell assays expounded that the declined migration and invasion of AKR1B10-silencing HSC-3 cells were both improved again after HK2 was overexpressed (Fig. 6A, B), which was further proved by the increased MMP2 and MMP9 protein expression in the siRNA-AKR1B10 + Ov-HK2 group relative to the siRNA-AKR1B10 + Ov-NC group (Fig. 6C). Consistently, AKR1B10 knockdown enhanced E-cadherin expression and reduced N-cadherin, Vimentin and Slug expression in HSC-3 cells. However, in AKR1B10-silencing HSC-3 cells, E-cadherin expression was decreased and N-cadherin, Vimentin and Slug expression were fortified again by up-regulation of HK2 (Fig. 7A–C). These data conform that AKR1B10 binds HK2 to drive the migration, invasion and EMT of OSCC cells.

## Discussion

Increasing evidence has well documented that the dysregulation of oncogenes or tumor suppressor genes is implicated in the occurrence and development of OSCC [1, 26], which has not been fully illustrated, however. Considering that the metastasis of OSCC is a complex and multistep process, the underlying molecular mechanism needs to be identified. In this study, AKR1B10 expression was discovered to be enhanced in OSCC tissues and cells and interference with AKR1B10 restrained the proliferation, migration, invasion, EMT as well as glycolysis of OSCC cells. Notably, AKR1B10 was revealed to bind with HK2 protein to contribute to the development of OSCC.

Recent literatures have shown that AKR1B10 is lowly expressed in gastric cancer [11], colorectal cancer [27] and adrenocortical carcinoma [28] while highly expressed in breast cancer [10] and hepatocellular carcinoma [29], and modulates the biological events in cancers including proliferation, migration and invasion, suggesting that AKR1B10 may act as a novel biomarker for cancers. Moreover, Huang et al. have determined AKR1B10 inhibitors as potential drugs for cancer treatment [30]. Notably, the elevated AKR1B10 expression in OSCC tissues has been also observed and is associated with tumor recurrence and poor prognosis [13–15]. Consistently, we also analyzed that AKR1B10 was greatly overexpressed in OSCC tissues and cells here. Loss-of-function experiments further proved that after AKR1B10 was knocked down, the proliferation, migration, invasion of HSC-3 cells were all extenuated, coupled with the depleted expressions of metastasis-associated proteins MMP2 and MMP9.

EMT is a cellular plasticity process featured by the conversion of the epithelial to the mesenchymal phenotype accompanied with the downregulation of epithelial markers such as E-cadherin and the upregulation of mesenchymal markers such as vimentin, N-cadherin and Slug [31]. Increasing evidence has unraveled that EMT is a crucial mechanism governing the initiation of migration, invasion and metastasis in OSCC [32, 33]. Furthermore, AKR1B10 also participates in the carcinogenesis and tumor development through regulating EMT in gastric cancer [11], hepatocellular carcinoma [29] and adrenocortical carcinoma [28] cells. Undoubtedly, our experimental results also clarified that AKR1B10 silencing raised E-cadherin expression while reduced N-cadherin, Vimentin and Slug expression in HSC-3 cells, implying the inhibitory role of AKR1B10 down-regulation in the EMT of OSCC cells.

Altered metabolism, which mediates many activities related to cell survival and fate, is one of the most striking characteristics of cancer cells [34]. As the foundation of cellular metabolism, aerobic glycolysis (also termed the Warburg effect) which is defined as the transformation of glucose to lactate along with the production of ATP, substitutes for the preferential use of glycolysis to provide energy independent of oxygen [35]. It is well established that through aerobic glycolysis, the metabolic demands for activities including EMT and metastasis in malignant tumor cells are met [36]. LDHA has been supported to activate the glycolytic pathway via irreversibly catalyzing the conversion of pyruvate to lactate [37]. PKM2 is a rate-limiting glycolytic enzyme that serves as an essential mediator of aerobic glycolysis [38]. HK2, one of the most pivotal glycolytic enzymes in the first rate-limiting step of glucose metabolism, mediates the phosphorylation of glucose to glucose-6-phosphate [17]. At the same time, emerging literatures have underlined that HK2 expression is boosted in OSCC tissues and cells and intensifies the aggressive phenotypes of tumor cells [21, 39]. Importantly, Cai et al. have proposed that METTL3 mediates the m6A modification of AKR1B10 to aggravate glycolysis and cholangiocarcinoma progression [24]. In the current paper, it was noted that deficiency of AKR1B10 inhibited ATP production, glucose consumption and ECAR and decreased HK2, PKM2 and LDHA expression in HSC-3 cells. Interestingly, the binding between AKR1B10 and HK2 was predicted by Monarchinitiative database, which was then validated by Co-IP assay. Further up-regulation of HK2 partially counteracted the impacts of AKR1B10 depletion on the proliferation, migration, invasion, EMT as well as glycolysis of HSC-3 cells.

## Conclusion

Overall, our study introduced the potential oncogenic role of AKR1B10 in OSCC through driving cell proliferation, migration, invasion, EMT and glycolysis functionally. Mechanistically, AKR1B10 protein bound glycolysis-related HK2 protein in OSCC cells. This finding implied that AKR1B10 might be deemed as a novel and potential therapeutic target for anti-metastatic and anti-glycolytic strategies for OSCC.

## Data Availability

The experimental data are available from the corresponding author on reasonable request.
